# Helping when the desire is low: Expectancy as a booster

**DOI:** 10.1007/s11031-020-09853-3

**Published:** 2020-09-04

**Authors:** Małgorzata Kossowska, Ewa Szumowska, Paulina Szwed, Aneta Czernatowicz-Kukuczka, Arie W. Kruglanski

**Affiliations:** 1 Faculty of Philosophy, Institute of Psychology, Jagiellonian University, Ingardena 6, 30-060 Kraków, Poland; 2 Faculty of Philosophy, Institute of Religious Studies, Jagiellonian University, Kraków, Poland; 3 Department of Psychology, Maryland University, College Park, USA

**Keywords:** Intention to help, Helping behavior, Motivation to help, Social judgment, Social cognition

## Abstract

One might assume that the desire to help (here described as *Want*) is the essential driver of helping declarations and/or behaviors. However, even if desire to help (*Want*) is low, intention to help may still occur if the expectancy regarding the perceived effectiveness of helping is high. We tested these predictions in a set of three experimental studies. In all three, we measured the desire to help (*Want*) and the *Expectancy* that the aid would be impactful for the victim; in addition, we manipulated *Expectancy* in Study 3. In Studies 1 and 3, we measured the participants’ declaration to help while in Study 2, their helping behavior was examined. In all three studies, we used variations of the same story about a victim. The results supported our hypothesis. Thus, the studies help to tease apart the determinants of helping under conditions of lowered desire to do so, an issue of great importance in public policymaking.

**Electronic supplementary material:**

The online version of this article (10.1007/s11031-020-09853-3) contains supplementary material, which is available to authorized users.

## Introduction

Vast research on helping focuses on the desire to help, i.e., the feeling of wanting to do something to help victims (for an overview see: Butts et al. 2019). However, eliciting a high desire to help is not always easy or even possible. This is usually the case in public policy decisions. Moreover, factors such as personal values, religion, personal experience (Charities Aid Foundation 2013), or demographic variables (Wunderink 2002) may lower people’s desire to help. Indeed, recent reports show that these factors systematically decrease the levels of charity in many countries (Charities Aid Foundation 2019; Roy Morgan’s Data 2019). Thus, the question arises as to how to foster helping in situations where the initial desire to do so is relatively weak.

In this paper, we build on motivational readiness theory (Kruglanski et al. 2014) by examining the role of *Expectancy* in making decisions about charity donations. *Expectancy* in this case can be defined as the subjective assessment of the extent to which any aid will be impactful for the victim. One might assume that the desire to help (here described as *Want*) is the essential driver of helping declarations and/or behaviors. However, even if *Want* is low, declared or actual help may still occur if the *Expectancy* regarding the perceived effectiveness of helping is high.

Although some researchers have implicated *Expectancy* as an influence on charitable-giving decisions (e.g., Bendapudi et al. 1996; Cryder et al. 2013a, b; Sharma and Morwitz 2016), their focus was on the separate (or additive) effects of affective (*Want*) and cognitive (*Expectancy*) responses to a person in need. In contrast to this, we demonstrate the interplay between these two factors, i.e., *Want* and *Expectancy,* via the results of three experimental studies presented in this paper. These help to tease apart the determinants of helping under conditions of lowered desire to do so, an issue of great importance in public policymaking, and in the creation of successful fundraising strategies of many charity organizations.

## What motivates helping?

Many researchers consider the desire to help victims as one of the most prominent motive driving declared or actual help (Hofmann and Dillen 2012; Butts et al. 2019). Desire is usually defined as an affectively charged motivation toward a certain object, person, or activity, whose satisfaction is associated with pleasure or a relief from displeasure (see Kavanagh et al. 2005). Indeed, different emotions, both negative and positive may accompany the desire to help and most psychological literature on helping focuses on analyzing these affective responses to people in need (e.g., Cialdini et al. 1987; Loewenstein and Small 2007; Batson 2011; Kogut and Ritov 2005; Genevsky et al. 2013; Cameron and Payne 2011; Butts et al. 2019).

On the other hand, studies have demonstrated that non-affective factors may play a role here, too. For example, people are more likely to help if they believe that their contribution will make a major impact (e.g., Bendapudi et al. 1996). Helping motivation decreases if the overhead costs are perceived as high (Sargeant and Woodliffe 2007), but increases when people believe the benefits of helping outweigh the costs (Erlandsson et al. 2014). This motivation to help also increases when campaigns align with people’s goals; in this circumstance, donors feel that their contribution will make a more substantial impact (Cryder et al. 2013a, b). Other research has shown that people donate more money when they believe they are the only possible helper who can assist an identified child as opposed to when the belief is that there is a shared responsibility to help all children (Basil et al. 2006; Cryder and Loewenstein 2012). Sharma and Morwitz (2016), distinguishing between perceived self-efficacy (i.e., the belief that one can take the steps required to achieve an outcome) and response efficacy (i.e., the belief that the steps taken will result in the desired outcome), demonstrated that increasing perceived self-efficacy increases perceived response efficacy, and donations to multiple beneficiaries. Also, recently, Touré-Tillery and Fishbach (2017) showed that the intention to help increases as the perceived impact of actions increases, such that the greater the expected impact of a contribution, that is, the greater the likelihood that the contribution will alleviate suffering, the more willingness there exists to take an action. In addition, Erlandsson et al. (2014) analyzed the role of perceived impact (also referred to as perceived utility or perceived efficacy) in explaining helping, showing that the higher the believed impact of the contribution, the more likely people are to help (see also Butts et al. 2019). All of the abovementioned results are in line with a survey conducted by the Charities Aid Foundation (2014); 72% of respondents declare that they ‘invest in charities who demonstrate their impact clearly’ and 81% of them believe that ‘more hard evidence of the impact of charities’ work’ would positively influence their decision to donate money.

### Interplay between affective and non-affective factors in motivating helping

In the studies mentioned above, researchers tested the affective and non-affective factors underlying help separately; we propose to look at the interplay between them. According to motivational readiness theory (Kruglanski et al. 2014), the willingness or inclination to act in the service of a desire is determined by two factors: *Want* and *Expectancy*. The *Want* state is defined as an outcome that a person desires at a given moment (e.g., a desire to help). *Expectancy* is a subjective assessment of the gratification of the *Want* (e.g., a subjective assessment of the extent to which aid will be impactful for the victim). Although both *Want* and *Expectancy* influence the inclination to act, they are not functionally equivalent in their effects. Certainly, desire (*Want*) seems critical and indispensable for undertaking any action. *Expectancy*, though also contributing to motivational readiness, according to the theory, plays more of an assisting role. The theory allows for the possibility of motivational readiness even where the *Expectancy* is absent. In contrast, if *Want* is entirely gone, no amount of *Expectancy* would suffice to rekindle one’s extinguished readiness.

It is worth stressing however that unlike in classic motivational models in which the *Want* and *Expectancy* factors are typically portrayed as fully independent from each other (e.g., Atkinson 1964; Hull 1943; Spence 1956; Tolman 1955), the motivational readiness theory indicated that they are in fact interdependent and that each may partially determine the other under some conditions (see also Dunning 1999; Kunda 1990; Kunda and Sinclair 1999). For example, at high degree of *Want* the individual may develop a hope of attainment even where objective *Expectancy* of attainment was low, demonstrating wishful thinking (McGuire 1960; Bélanger et al. 2016). Similar “motivated distortions” were discussed also in other areas and suggest that when people really want something, they find it more achievable and likely (or unlikely in case of something they find highly undesirable, see Kruglanski 1999; Kruglanski and Ajzen 1983). The higher the *Want*, the greater the tendency to distort reality, or subordinate it to the dominant desire. Nonetheless, the *Want* factor has major determinants other than expectancy, for example the degree of deprivation (as in hunger) or magnitude of the incentive (e.g. the tastiness of the food). Thus, we expect that *Want* and *Expectancy* even though partially linked, are still largely independent of each other, hence they constitute mostly separate sources of behavioral intentions.

In summary then, we assume that *Want* is a fundamental factor in predicting declared or actual help. If desire is at a high level, *Expectancy* is not essential to take an action, as long as it reaches a minimal threshold level. However, if *Want* to help others is flagging, declared or actual help may still occur when prompted by high degrees of *Expectancy.* In such a circumstance (i.e., low *Want*, high *Expectancy*), the magnitude of readiness to help is smaller compared to the situation of high *Want*, due to the crucial role of the latter; however, the declared or actual help should still be exhibited.

## Overview of the studies

We tested the foregoing predictions in a set of three experimental studies.^1^ In all three, we measured the desire to help (*Want*) and the *Expectancy* that the aid would be impactful for the victim; in addition, we manipulated *Expectancy* in Study 3. In Studies 1 and 3, we measured the participants’ declaration of their intention to help. We are aware that desire to help (*Want*) and declaration/intention to help might be seen as difficult to distinguish. It is because within attitude theory the concepts of desires and intentions are not differentiated but are often treated as synonyms. However, Perugini and Bagozzi (2004) demonstrated that there are theoretical reasons for distinguishing between desires and intentions. Also, an emerging literature shows that desires strongly influence intentions and substantially mediate most of the effects of attitudes, subjective norms, perceived behavioral control, and other personal reasons for acting on intentions (e.g. Bagozzi and Edwards 1998a, b). Trying to distinguish between desire and intention, Perugini and Bagozzi (2004) indicate that intentions have a stronger connection to goals or outcomes than desires because they imply a commitment, and encompass at least some form of partial planning to achieve the goals or outcomes (Bratman 1987), whereas desires do not. The emphasis on *goals*, representing combinations of *Wants* and *Expectancies* have inspired recent advances in the theory of reasoned action (see, Kruglanski et al. 2016; Ajzen and Kruglanski 2019).

Intentions to act are directly connected to a multitude of activities and outcomes related to the choice of means for action implementation, impediments to action, temptations to perform other actions or consider other goals, cues for retrieval of the intention at a future point in time, and so forth (Bagozzi and Edwards 2000). Also, within the motivational readiness theory the two constructs can be distinguished. *Want* produces motivational readiness, however, intention is formed only after the commitment point is passed, that is a goal is formed. For the latter, a minimum level of *Expectancy* is also needed. For instance, one might desire to climb Mount Everest, however, they may form the intention to do so (and actually climb it) only when they find it feasible (i.e. expectancy of success is above threshold), hence committing to the goal to climb.

Our three studies contained common elements allowing convergence but also contained unique features that complemented each other. Whereas in Study 1 we examined behavioral intentions in Study 2 we additionally examined participants helping behavior, instead of mere declarations of intention. In Studies 1 and 3, we also measured negative affect. As we did not have specific predictions about its impact, the results for this variable are presented in Supplementary Materials.

In all three studies, we used variations of the same story about a victim. The basic version of the story read as follows:

A 5-year old boy/girl, who suffers from a serious kidney condition, is now in hospital. The condition will soon lead to kidney failure, which will put the boy’s/girl’s life in danger. For medical reasons, a kidney transplant is impossible as is hemodialysis. However, a medication which can stop the disease has been recently discovered. Unfortunately, health insurance will not pay for it in Poland, and the treatment is very expensive. If the amount of 500,000 PLN is not collected shortly, the progress of the disease will be so advanced that it will be impossible to save the boy/girl.

In all our studies, we tested the interactive effect of the desire to help (*Want*) and *Expectancy*, and hypothesized that, at high levels of *Want*, *Expectancy* would not matter; that is, the desire to help would be the sole significant predictor of the magnitude of the (intended) help. At the same time, we also hypothesized that at lower levels of *Want*, *Expectancy* would significantly predict helping declarations and behaviors.

In order to determine the samples size, we ran an a priori power analysis with the use of G*Power 3.1 (Faul et al. 2009). As for estimates of effect sizes in Studies 1 and 3, they were based on what is typically done in the discipline when the effect size is unknown (i.e. medium effect size is assumed). Since in our studies we expected a significant effect of *Expectancy* only at low levels of *Want*, this is where we assumed a medium effect size (β = 0.5). At high levels of *Want* we assumed no significant effect of *Expectancy* (effect equal to β = 0), so the expected difference between conditions was equal to 0.5. Such a difference produces an effect size of f^2^ = 0.07 for the interaction (rather small effect). This is what we entered in our analyses. In Study 2, we also assumed a rather small effect for the whole interaction (odds ratio equals to 2). In all studies, we strived for achieving at least 0.90 power.

The Local Research Ethics Committee approved all studies. Thus, data collection complied with current APA Ethical Principles of Psychologists and Code of Conduct. The data is available at https://osf.io/g5pe6/?view_only=375446b4157a4823b814a8505c191825.

## Method

### Study 1

In Study 1, we checked if increased *Expectancy* predicts declarations regarding readiness to help even when the desire to help is low. We thus ran an online study in which we presented participants with a helping scenario and recorded their declared intentions to help. Their desire to help (*Want*) and the *Expectancy* that the help would be impactful were measured as well. We investigated whether the interaction between the two factors predicts the declared magnitude of the help.

#### Participants

A priori power analysis with the use of G*Power 3.1 (Faul et al. 2009) showed that a sample of at least 153 would be necessary to obtain the assumed power. Since the study was conducted online, we increased the sample size by 30% to take into account possible attritions and non-attentive responding. Accordingly, we aimed to recruit at least 204 participants for this study.

Two hundred and nine registered users of the Research Online platform took part in the online experiment. There were 126 women and 83 men, aged between 18 and 84 (*M* = 33.94, *SD* = 11.35). Sixty-eight participants had a high school education, and 5 had occupational education; 21 were students, and 115 had completed their higher education at the time of the study. Participants were paid for their participation in line with the rules of the platform (approx. 2 EUR). All participants gave informed consent before participation in the study, and they were debriefed on completion of the study.

Fourteen participants failed our attention check (see below). Therefore, the final sample consisted of *N* = 195 participants (116 women, 79 men; with a mean of age *M* = 33.93, *SD* = 10.85).

#### Materials and procedure

In the study, we presented participants with a story of a child, or children, in need (it transpired that the version of the story was unimportant, see Supplementary Materials). Then, the participants’ desire to help, and expectancy in relation to the satisfaction of this desire, were measured. Want was measured using one item: *To what extent is it important for you to help [the victim]?* (1–7 scale anchored with *not at all important* and *definitely important*). We also used an additional item: *Do you want to help in this situation?* But we decided not to include it to the analysis as it is linked too close to measure of intention. We however re-analyzed data using both items and the results were the same. Alongside this, *Expectancy*^2^ (Cronbach’s *α* = 0.81, *M* = 5.08, *SD* = 0.93) was measured with four items: (1) *In your opinion, by donating, will you effectively help [the victim]?* (2) *In your opinion, will the necessary amount be collected?* (3) *In your opinion, if the necessary amount is collected, will it be possible to save…?* (4) *In your opinion, are actions like this effective?* (1–7 Scale anchored with *definitely not* and *absolutely*). Indices of *Want* and *Expectancy* were obtained by averaging responses to the respective items. The order of *Want* and *Expectancy* items was counterbalanced.

Finally, participants were asked about their donation declarations with the following question: *What amount would you be willing to donate to this cause?* (Participants were asked to mark an amount on a slider). The minimum amount was 0 PLN, and the maximum was 100 PLN, with responses possible in 1 PLN increments. We decided to set an upper limit to make sure that all participants operated within a given range and that their donation decisions would not be influenced by other factors (e.g., their socio-economic status).

In addition, we decided to control for the participants’ gender and attitude towards money as these variables may influence the declarations to donate or actual donation. Indeed, some research show that women are likely to give and give more than man in similar situations (Mesch et al. 2011; Cox and Deck 2006; Anderoni and Vesterlund 2001). Besides, it is already known that people differ in their attitudes towards money, that money attitudes are mostly independent from income, and money perceptions are an additional important factor in the understanding of charitable behavior (Wiepking and Breeze 2011). Thus, even though participants did not donate actual money, their decisions could be influenced by their attitude towards spending. Therefore, we asked participants to rate to what extent they agreed with the following statements: (1) *I attach a lot of importance to money*. (2) *I appreciate the value of money*. (3) *I carefully think about each zloty spent*. (4) *Money is important in my life* (1–7 scale from *definitely disagree* to *definitely agree*). Responses to all items were averaged and the resulting score was entered into the analyses as a statistical control (reported in the Supplementary Materials).

Since the study was run online, we also included an attention check. Specifically, we added a question in which we asked participants to mark response number 3.

#### Results and discussion

Means and intercorrelations between variables are presented in Table 1. As *Want* and *Expectancy* are correlated, we explored the variance inflation factor (VIF) to be sure that performing interaction is accurate (Freund et al. 2003). As *VIF* equals 1.37, we performed interaction analysis. To test the interactional hypothesis, we regressed *Want*, *Expectancy*, and the product of the two on the donation (log transformed prior to the analyses due to the skewness in the data, DV’s skewness = 1.28). The analyses were performed with the use of Process macro for SPSS version 2.13 (Hayes 2013). In all analyses, bias corrected bootstrap confidence intervals are reported; 10,000 bootstrap samples were used in all analyses. All variables were standardized prior to analyses.

**Table 1:** Descriptive statistics and intercorrelations

	M	SD	1	2
Study 1 (N = 195)				
(1) Want [1–7]	4.81	1.31		
(2) Expectancy [1–7]	5.08	0.93	.48*	
(3) Money donated [1–100 PLN]	27.01	26.07	.49*	.36*
Study 2 (N = 134)				
(1) Want [1–7]	4.63	1.25		
(2) Expectancy [1–7]	5.02	1.35	.44*	
(3) Money donated [1–20 PLN]	3.62	4.72	.37*	.26*
Study 3 (N = 190)				
(1) Want [1–7]	4.71	1.71		
(2) Expectancy [1–7]	5.13	1.13	.65*	
(3) Money donated [1–100 PLN]	26.11	24.14	.50*	.34*

^*^Significant

**Fig. 1:**
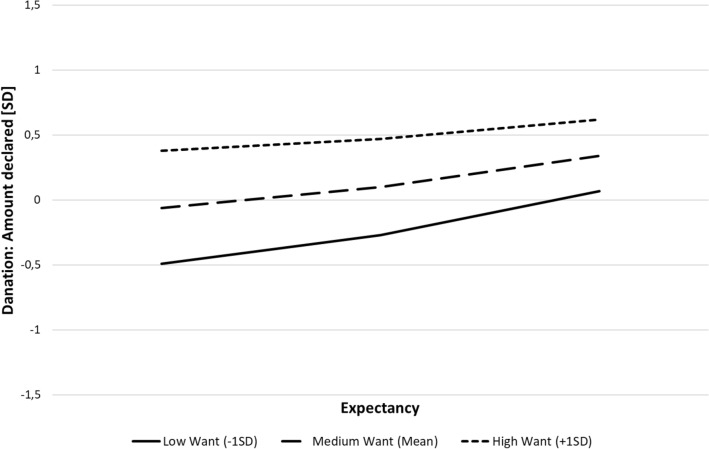
Interactive effect of *Want* and *Expectancy* on donation (Study 1)

The results showed that both *Want* and *Expectancy* significantly predicted declarations to donate, *β* = 0.48, *t* = 7.26, *p* < 0.001, 95% CI [0.35, 0.61] for *Want*, and *β* = 0.21, *t* = 3.23, *p* = 0.001, 95% CI [0.08, 0.34], for *Expectancy*. Importantly, however, there was a significant interaction, *β* =  − 0.10, *t* =  − 2.10, *p* = 0.04, 95% CI [− 0.19, 0.00]. Conditional effects analysis showed that, whereas there were no significant effects of *Expectancy* at high (+ 1 *SD*) values of *Want*, *β* = 0.12, *t* = 1.45, *p* = 0.15, 95% CI [− 0.04, 0.28], there was a significant effect of *Expectancy* at low (− 1 *SD*) values of *Want*, *β* = 0.27, *t* = 3.94, *p* < 0.001, 95% CI [0.14, 0.41]. The interaction is graphically presented in Fig. 1.

We re-ran the analyses while controlling for gender and attitudes towards money, and they yielded similar results (see Table 1 in Supplementary Materials).

The results thus demonstrated that, although a desire to help (*Want*) is a crucial factor influencing on declarations to donate, if it is low, *Expectancy* that the donation is effective matters as well. Specifically, when the desire to help was high, *Expectancy* did not seem to affect the donation. However, when the desire to help was low, *Expectancy* became a significant predictor, with high *Expectancy* levels significantly increasing the declared amount.

### Study 2

The aim of Study 2 was to replicate the results described above in a laboratory setting. In addition, we also focused on actual helping behavior, not merely on declarations to donate as in Study 1. It is important extension of Study 1 as declarations made by people about how they behave might not always be consistent with their overt behavior (see Doliński 2018). Thus, it is crucial to demonstrate that our predictions hold also when actual helping behavior is taken into account. Therefore, we set up a laboratory experiment connected with a charity fundraiser in which participants could donate money earned in the lab to a person in need. As in the previous study, *Want* and *Expectancy* were measured, and we tested whether they interactively predicted participation in the fundraiser (i.e., in the form of offering help).

#### Participants

We ran an a priori power analysis for logistic regression with a power of 0.90 and odds ratio of 2. The analysis showed that a sample size of at least 106 participants would be necessary to obtain the assumed power. We intended to recruit more participants in case there were any data losses; we therefore endeavored to enroll at minimum 130 participants in the study.

We recruited 134 participants to take part in a laboratory study on solving cognitive tasks. The sample was comprised of young adults who answered an announcement at university websites and local internet portals. There were 111 women and 21 men (2 participants did not provide their demographics) and they were aged between 18 and 35 (*M* = 23.66, *SD* = 3.54). 63 Participants had received a high school education; 2 had occupational education, 2 basic education, and 65 higher education. 50 Participants were students, 20 were employed, 44 both studied and worked, and 18 were doing neither at the time of the experiment.

Participants were paid 20 PLN (around 5 Euro) for participation in the study. All subjects gave informed consent before participation in the study and were debriefed on its completion.

#### Materials and procedure

Participants were recruited to take part in a study on task solving. In this study, which took about an hour to complete, they solved a set of computer tasks of low to medium difficulty. Participants worked in sessions of up to six participants at the same time, each in a separate cubicle. After completing the task, they were paid the amount of 20 PLN (in one zloty coins; app. 4.5 Euro) and thanked for participation in the study. However, they were also informed that the study was run in cooperation with a local university charity organization which was currently raising money to help children in need. Each participant was presented with a description of the cause in writing, i.e., they read about a child suffering from a kidney disease (in one of the three versions, as presented in the Supplementary Materials; the version did not matter).

Participants were informed that if they decided to support the cause, their money would be given to the charity in full. They were also told that the fund-raiser was not part of the study and that donations were entirely voluntary so their decision to take part was entirely up to them. Nevertheless, all participants were asked to answer several questions, allegedly for evaluation purposes, even if they decided not to donate any money. The questionnaire aimed to measure the desire to help (*Want*), the expectancy that this desire would be satisfied (*Expectancy*)*,* and to assess their attitude towards money. Since we wanted the participants to believe this was not a part of a study, we kept the questionnaire short and asked one question per each variable: *To what extent is it important for you to help the child?*—To measure *Want*; *In your opinion, are campaigns like this effective?*—To measure *Expectancy*; and *I carefully think about each zloty spent*—to measure their attitude towards money (each responded on a 1–7 scale).

Participants answered the questions in their cubicles so that they could have some privacy and not feel pressured in any way. After answering the questions, they were asked to put the questionnaire, along with the amount (if any) they were willing to donate, in the envelope and leave it on the desk (as participants were paid in single zloty coins, they could leave any amount ranging from 1 to 20 PLN). After completing the study, they were informed that they would soon receive detailed information regarding the purpose of the study and confirmation of their financial donation to the charity organization via email.

In the follow-up email, the purpose of the study was explained to them, and they were provided with more information about the charity organization to which the money was donated (the organization was the Association for Aid to Children with Kidney Diseases associated under the auspice of Professor Marta Uszycka-Karcz, www.nefrologiadziecieca.pl). A receipt for the money transfer was also attached.

#### Results and discussion

Means and intercorrelations between variables measured in this study are presented in Tables 1 and 2. As *Want* and *Expectancy* are correlated, we explored *VIF* index to be sure that performing interaction is accurate. As *VIF* equals 1.22, we performed interaction analysis.

**Table 2:** Mean differences for variables tested in Study 2 depending on decision to donate (N = 134)

	M	SD	t	p-value
Want				
No donation	4.08	1.41	− 3.88	< .001
Donation	4.96	1.01		
Expectancy				
No donation	4.61	1.60	− 2.5	.015
Donation	5.26	1.12		

Since it has been argued that observed behavior, unlike declarations, is often binary, and that it is the decision to donate or not that is of crucial importance (Doliński 2018), we analyzed the effects of *Want* and *Expectancy* on the decision to help. To that end, we ran logistic regression on the decision to help (0 when a participant did not donate anything, 1 if they donated something).

**Fig. 2:**
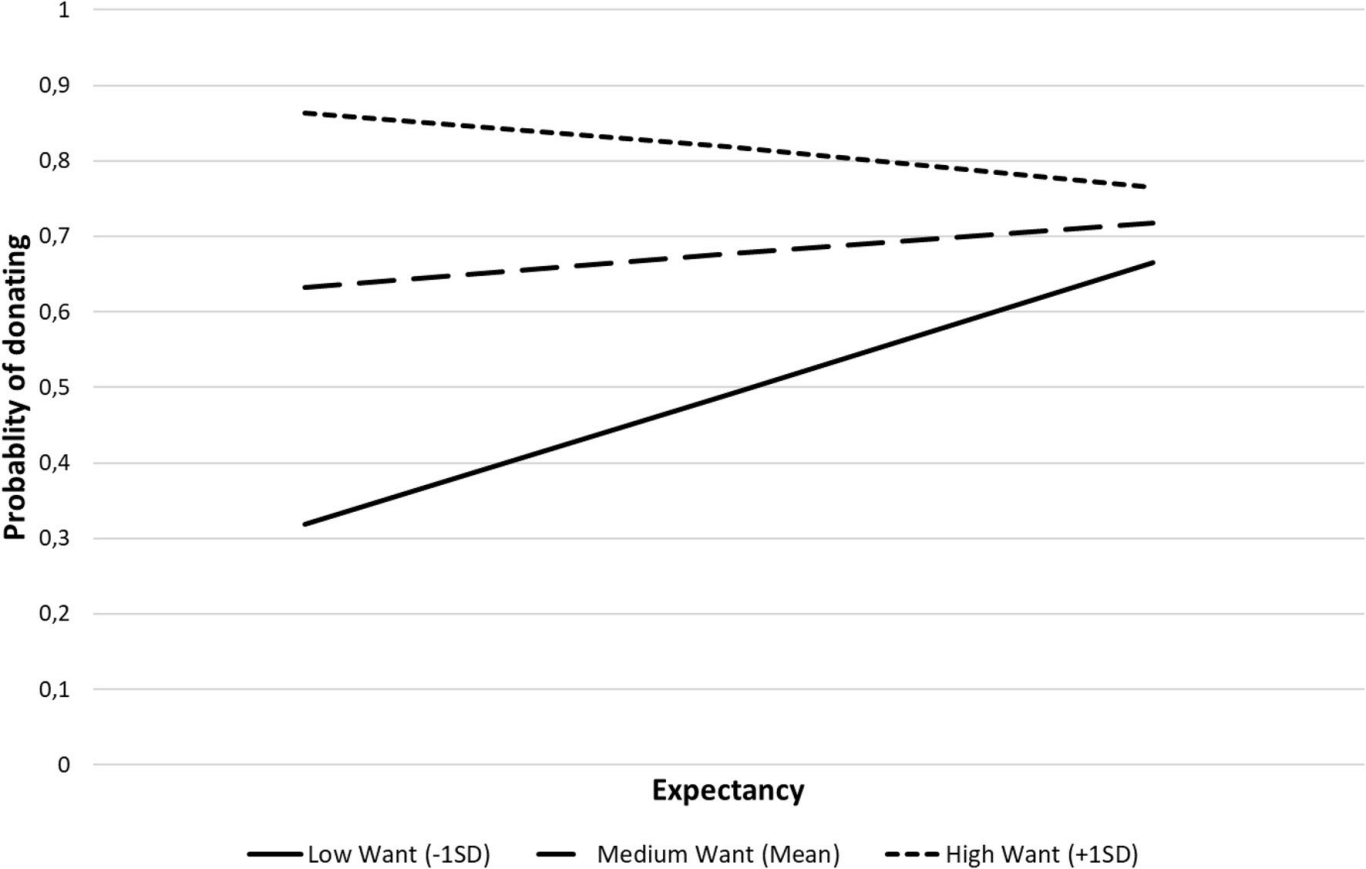
Probability of donating depending on *Expectancy* at different level of *want* (Study 2)

The results showed that there was a significant effect of *Want*, b = 0.77, *Z* = 2.96, *p* = 0.003, 95% CI [0.27, 1.28], whereas *Expectancy* was non-significant, b = 0.20, *Z* = 0.86, *p* = 0.390, 95% CI [− 0.25, 0.65]. Importantly, however, there was a non-significant *Want* × *Expectancy* interaction, b =  − 0.53, *Z* =  − 1.86, *p* = 0.063, 95% CI [− 1.08, 0.03]. Conditional effects analysis showed that, whereas there were no significant effects of *Expectancy* at high (+ 1 *SD*) values of *Want*, b =  − 0.33, *Z* =  − 0.91, *p* = 0.364, 95% CI [− 1.05, 0.39], there was a significant effect of *Expectancy* at low values of the moderator, b = 0.72, *Z* = 1.99, *p* = 0.047, 95% CI [0.01, 1.44]. The interaction is graphically presented in Fig. 2. Similar results were obtained when controlling for gender and attitudes towards money (see Table 11 in Supplementary Materials).

To test if *Want* and *Expectancy* predicts the sum of donation (log transformed prior to the analyses due to the skewness in the data, skewness = 1.58) we ran similar analyses as we did in Study 1. The results showed that there was no interactional effect of *Want* and *Expectancy* on donation, *β* =  − 0.02, *t* =  − 0.32, *p* = 0.748, 95% CI [− 0.16, 0.12]. *Want* was a significant predictor of donation, *β* = 0.35, *t* = 3.93, *p* < 0.001, 95% CI [0.17, 0.53], whereas *Expectancy* was not, *β* = 0.10, *t* = 1.08, *p* = 0.280, 95% CI [− 0.08, 0.28].

Thus, we demonstrated that, although it was the desire to help that mainly predicted helping behavior, when it was low, high *Expectancy* predicted donations as well. The study adds to the previous one by showing that the interactional effect of *Want* and *Expectancy* not only predicts declarations, but also the actual behavior subsequently displayed. Unlike in the previous study, we didn’t find significant effects on the amount of money donated, which might stem from the fact that this time we asked participants for real donations not just declarations. It is in line with the results of other studies investigating actual helping behaviors (vs. declarations), showing that the decision whether to donate differs from the decision on how much to donate (Parsons 2007; Karlan and Wood 2017; Bergh and Reinstein 2020).

### Study 3

In Study 3, to more directly test if increased *Expectancy* may predict donations if desire to help is low*,* we manipulated the level of *Expectancy* by providing participants with information about the chances of success of collecting the money for the given victim*.*

#### Participants

We calculated power for this study in a similar manner to that carried out for Study 1 but the interaction was calculated for the high and low *Expectancy* conditions rather than for high and low values of the moderator. The analysis yielded similar results and we aimed to recruit 204 participants for this study.

The sample was comprised of 205 participants. There were 167 women and 38 men ranging in age from 18 to 44 (*M* = 23.19, *SD* = 3.91). All participants were enrolled in a lottery in which they could win three prizes of 50 PLN each. All subjects gave informed consent before participation in the study and were debriefed upon completion of the study.

Fifteen participants failed our attention check (see below). Therefore, the final sample comprised *N* = 190 participants (156 women, 34 men; with the mean age *M* = 23.06, *SD* = 3.68).

#### Materials and procedure

Participants read a story about a child (or children) in need. Additionally, we manipulated the *Expectancy* of the fund-raiser’s success by adding information about the amount of money that had been collected so far. One group of participants was told that: *The money collection is ending soon and, so far, only 50,000 PLN has been collected*; this was the low *Expectancy* condition. Another group of participants was told: *The money collection is ending soon and, so far, 450,000 PLN has already been collected*; this was the high *Expectancy* condition. All participants were given the information that 500,000 PLN needed to be raised. We assumed that by informing participants about the progress of the fund-raiser, we would influence their expectations of success regarding the whole campaign. The manipulations used in each condition are presented in the Supplementary Materials.

We used the same measures of *Want*, *Expectancy* (*Cronbach*’*s α* = 0.75, *M* = 5.13, *SD* = 1.13), and donations as in Study 1. The participants’ attitudes toward money were also measured. An attention check question was also included (participants were asked to mark answer number 4).

#### Results and discussion

Descriptive statistics and intercorrelations between the variables are presented in Table 1. As *Want* and *Expectancy* are correlated, we explored *VIF* index to be sure that performing interaction is accurate. *VIF* equals 1.74, thus we performed analysis.

As predicted, the manipulation significantly affected *Expectancy*, *F*(1, 188) = 11.52, *p* = 0.001, partial *η*^2^ = 0.06 (*M* = 4.84 in the low and *M* = 5.38 in the high *Expectancy* condition). There was no effect on *Want*, *F*(1, 188) = 0.80, *p* = 0.372.

**Fig. 3:**
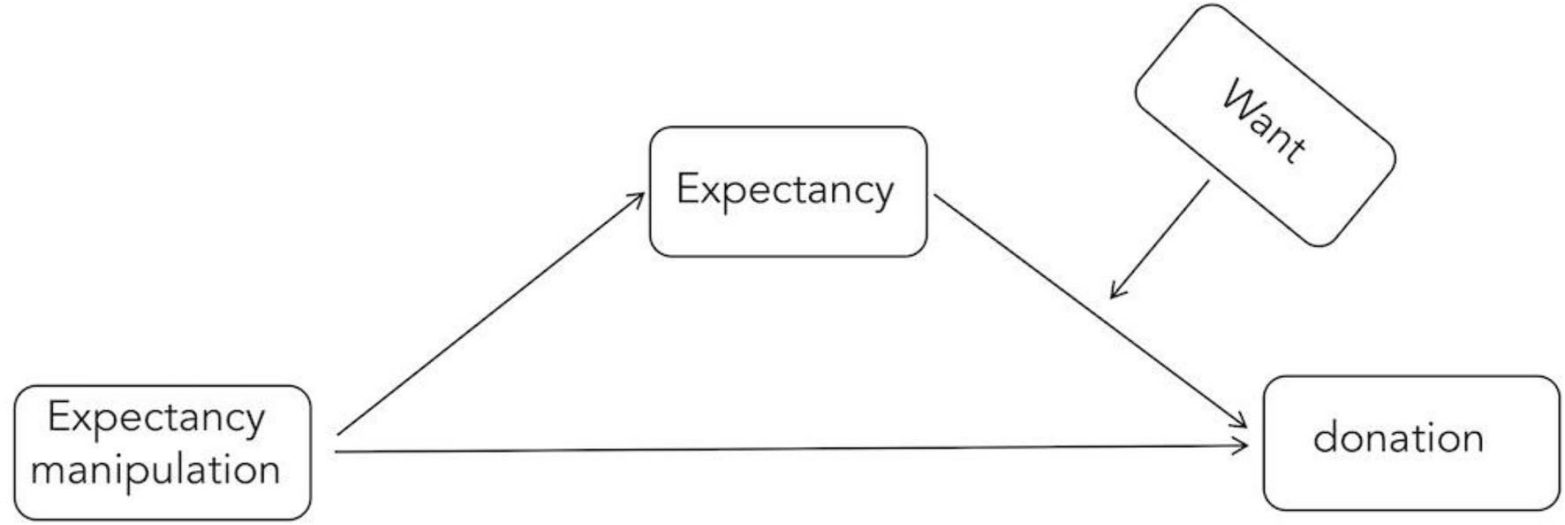
Model tested in Study 3

Next, we tested whether our manipulation significantly predicted the magnitude of the donation (log transformed prior to the analyses due to the skewness in the data, skewness = 1.16) via *Expectancy* at different levels of *Want*. We thus tested a moderated mediation model (Hayes 2013, model 14), with the manipulation as the IV, *Expectancy* as the mediator, *Want* as the moderator, and the donation as the DV (the model is pictured in Fig. 3). As in Study 1, the variables were log transformed prior to the analyses due to the skewness in the data. The analyses were performed with the use of Process macro for SPSS version 2.13 (Hayes 2013). In all analyses, bias corrected bootstrap confidence intervals are reported; 10,000 bootstrap samples were used in all analyses. All variables were standardized prior to analyses.

**Fig. 4:**
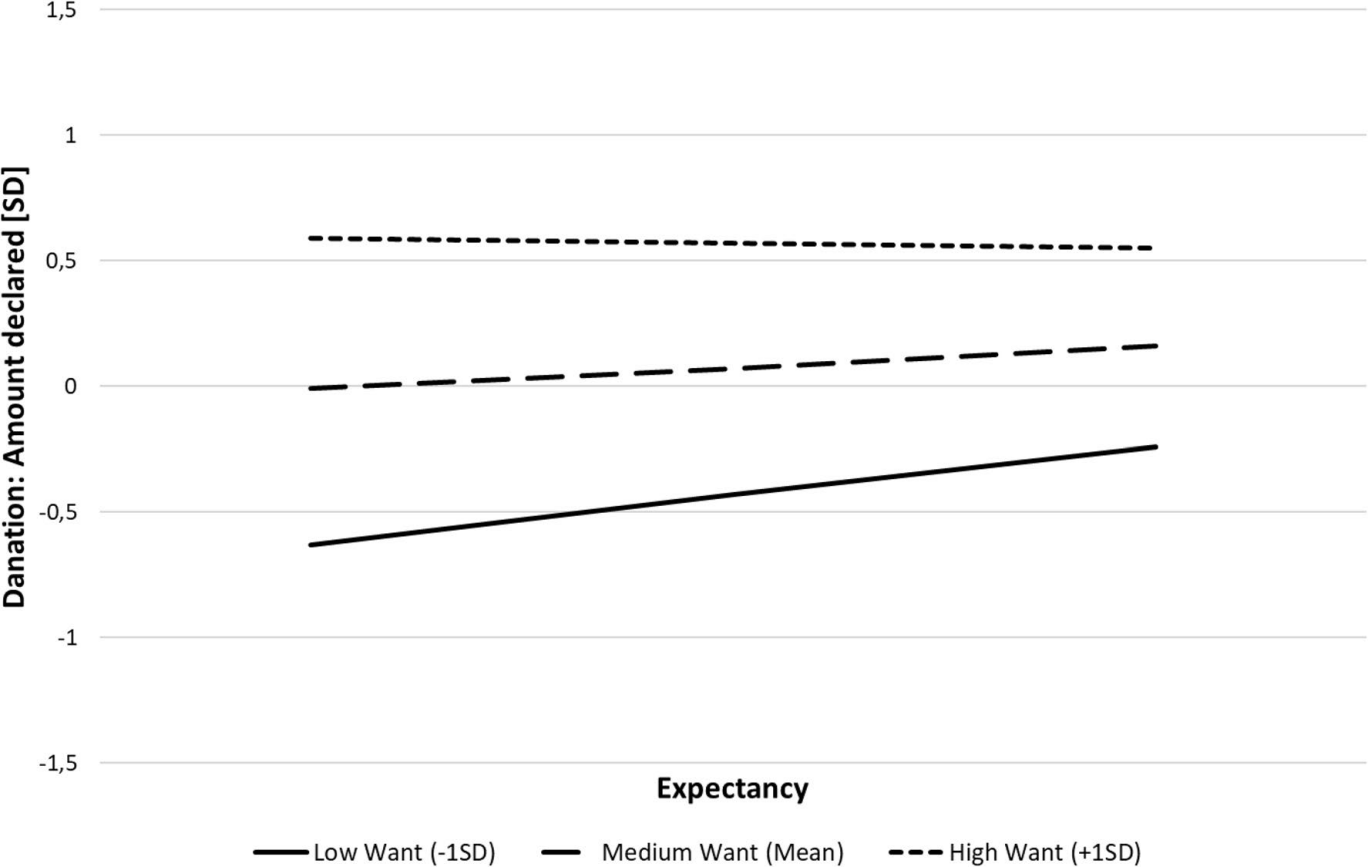
Interactive effect of *Want* and *Expectancy* on donation (Study 3)

The results showed a significant moderated mediation effect, *IMM* =  − 0.06, SE = 0.03, 95% CI [− 0.14, − 0.01], with the manipulation significantly predicting donations at low levels of *Want*, *IE* = 0.11, SE = 0.06, 95% CI [0.02, 0.27]. The effect was not significant at high values of *Want*, *IE* =  − 0.004, SE = 0.06, 95% CI [− 0.12, 0.12], suggesting that when *Want* was high, it was of no consequence what our participants were told about the likelihood of collecting the necessary amount. The *Want* × *Expectancy* interaction is presented in Fig. 4.

Thus, this study replicated our previous findings and provided further support for the interactive influence of *Want* and *Expectancy* on the declaration of donating. As in previous studies, *Want* was the main predictor of declarations to help, and when it was high, the level of *Expectancy* was immaterial. However, when *Want* was low, the amount of declared donations significantly increased with increasing *Expectancy*. Moreover, we showed that experimentally manipulating *Expectancy* translated into an increased willingness to help among participants who were not otherwise inclined to do so (i.e., those feeling low levels of *Want*).

### Summary of the results

To present the results obtained in a more illustrative manner, we statistically summarized the results of Studies 1, 2, and 3. The summary of the study results was performed in Comprehensive Meta-Analysis Software (CMA; Borenstein et al. 2013). We used Pearson’s *r* as a measure of the effect size and the sample size to calculate sampling variance. We fitted a fixed-effects model with the level of *Want* as a moderator. The heterogeneity among the effects was significant [*I*^2^ = 60.86%; *Q*(5) = 12.76, *p* = 0.003] and expected, given the differences in studies designs.

**Fig. 5:**
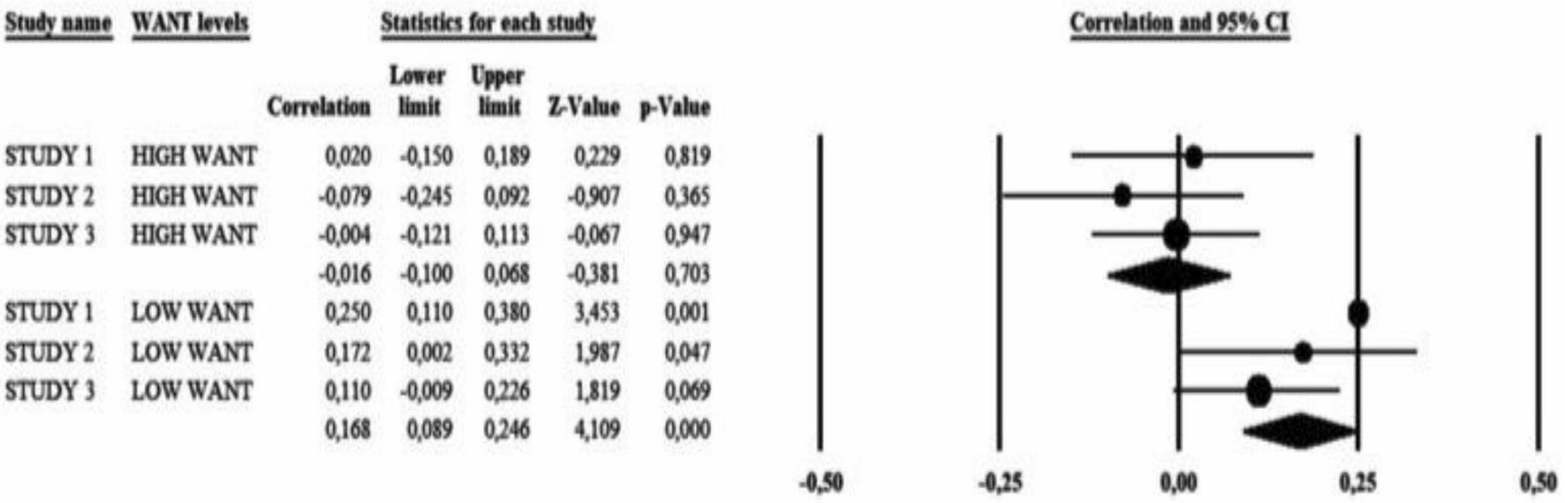
Summarization of Studies 1, 2 and 3 using meta-regression technique. *Note* in Study 2 the decision to donate (not the decision how much to donate) was included into the analysis

The results showed that the overall effect for the three studies followed the same pattern as the individual studies. To be specific, *Expectancy* was not associated with helping behaviors when *Want* levels were high (*r* =  − 0.02, *p* = 0.703, 95% CI [− 0.10, 0.07]). However, it was significant and positive at lower levels of *Want* (*r* = 0.17, *p* < 0.001, 95% CI [0.09, 0.25]). These results are summarized in Fig. 5.

## General discussion

The overall goal of this study was to gain a better understanding of the psychological underpinnings associated with helping people in need. We built on motivational readiness theory (Kruglanski et al. 2014) to test the role of *Expectancy* regarding the perceived effectiveness of helping in predicting helping declarations as well as behavior. In line with the theoretical assumptions, we systematically demonstrated that, although a desire to help (*Want*) is the essential driver of helping, when this factor is low, both helping declarations (and behaviors) may still occur if the *Expectancy* is high that the aid provided will be impactful for the victim(s). Despite the fact that a similar notion was previously investigated by other researchers (e.g., Sargeant and Woodliffe 2007; Erlandsson et al. 2014; Cryder et al. 2013a, b; Sharma and Morwitz 2016), in those studies, it was suggested that separate mechanisms underlay the act of helping a victim—with emotional mechanisms being responsible for helping identified victims, and cognitive mechanisms being responsible for helping statistical victims. We however have demonstrated that both components are essential in predicting declared or actual help with the desire to help (*Want*) being the pivotal predictor of helping, irrespective of the levels of *Expectancy* that the helping action will be effective. However, when the desire to help (*Want*) is low, helping is still possible via high *Expectancy*. To the best of our knowledge, this is the first systematic demonstration of an interactive effect of cognitive and affective components in predicting helping intentions and behaviors.

However, we should also note that, although we treat *Want* and *Expectancy* as separate mechanisms, these factors are not independent. This line of thought is in accordance with the motivational readiness theory which assumes that a relation between the two components might exist. This is exemplified in ‘wishful thinking’: when a person really wants something and thus perceives it as more attainable, or when a person’s desire increases when they perceive something as easily attainable or readily available. Such a relation is also seen in our studies, in which we found a moderate correlation between the two variables (see also Caviola et al. 2020). The correlations, however, suggest that these are still two distinct variables, and our further analyses show that they differentially predict helping motivation and behavior.

It is worth noting that in Study 2, although we found the expected effects on decision *whether to donate*, we didn’t find them on decision *how much to donate*. As mentioned above, it is consistent with other studies on actual donations, which demonstrated that the decision to donate differs from the decision on amount of the donation (Parsons 2007; Karlan and Wood 2017; Bergh and Reinstein 2020). Also, Dickert et al. (2011) suggest that donation decisions should be regarded as two-stage process, in which the initial decision to donate (stage 1) is separated from the donation amounts (stage 2). Authors suggested that different emotional mechanisms govern each stage (mood management in stage 1 and empathy in stage 2). However, in case of real (vs. hypothetical) donations other factors may play a role as well. As CAF’s report suggests (Charities Aid Foundation 2019), over half of people donate money for altruistic purposes, but the amount of money given was influenced by one’s personal values, sense of morality, faith, etc. (Charity Aid Foundation 2014). Given that we did not focus on the individual differences in our studies (except from attitude toward money), we were able to detect an effect in stages 1 and 2 in case of hypothetical donations, which are in fact only declarations, but not in case of real donations. In addition, we asked for donations only people who decided to donate (of 130 participants, 50 didn’t offer anything), which significantly reduced the sample size. Thus, it is possible that for this analysis our study was underpowered. Also, the variation of our dependent variable was quite small. Finally, there were differences between the format of the scales: in Study 1 the scales ranged between 0 and 100, but in Study 2 between 0 and 20. It means, that in Study 2 the variation of the results was lower than in Study 1.

There are limitations to the research reported here. One limitation is that we used self-reported measures of desire to help (*Want*), and expectancy to satisfy this desire*.* Such measures always decrease the ecological validity of the studies, and because many scholars believe they exhibit less construct validity, they should be used with caution (Kuncel et al. 2005)*.* In future studies, it would be beneficial to focus on more implicit measures—behavioral or psychophysiological. It is also worth noting that we conceptualize *Want* in a rather general fashion, as the importance of helping. However, the desire to help might be determined by a variety of factors (e.g. sympathy, empathy, the desire to be perceived in a positive manner) which tap into the affective component to help. In future studies, it would be of benefit if *Want* was operationalized in a different way, with different sources of *Want* being measured.

Another limitation may reside in the fact that our research does not focus on the emotional mechanism underlying the desire to help. Thus, we simply demonstrated that in the case of our manipulation (the invariable cause of the victim’s plight was a disease) negative affect is related to the desire to help. As emotional reactions to victims may be influenced by the particular circumstances that victims are facing, future research should investigate emotional responses to victims experiencing various sorts of plights to corroborate the results of our studies. Besides, we also think that it is possible that after answering the questions about *Want* and *Expectancy*, participants would respond highly to giving/donating due to social desirability. Indeed, the participants presented high willingness to help (see the skewness of the donation variable). In the future studies, the role of social desirability needs to be tested to rule out this possibility. Finally, we believe that an additional study with manipulation of *Want* and *Expectancy* separately might make our argument stronger. There are two reasons however why we were reluctant to run additional study. First, in Studies 1 and 2 we tried to manipulate *Want* via identifiability. Above we discuss why this manipulation didn’t work and suggest that future research should investigate emotional responses to victims experiencing various sorts of plights. To meet this goal, we would have to change the procedure of the study completely, thus the new study wouldn’t fit to the present series of the studies. In fact, in the meantime we did additional study with completely different procedure when we manipulate *Want*. Although this time the manipulation worked, and we replicate the previous results, we decided not to include this study into the paper to keep this paper more concise. However, we included this study into the Supplementary Material (see Study 0). Second, we also think that running the study about helping in COVID-19 situation can heavily influence the results (e.g., the helping mindset might be currently more salient than before). Thus, the replication of the previous results using exact the same procedures wouldn’t be possible and the results wouldn’t be comparable with the previous studies as the social context has changed drastically*.*

From a pragmatic standpoint, our results suggest ways of designing an effective charitable fundraising or crisis relief campaigns, that is, campaigns that increase the audience’s willingness to donate to victims or bodies tasked with handling the crisis (e.g. COVID-19 pandemic). Our results suggest that the message should address the prospects of goal attainment if just evoking emotions with regards to the victim is not possible. Past studies have suggested that presenting the victim by means of a photo depicting the victim’s circumstances (e.g., a photo of a hungry child expressing sadness) or with a specific background story about him/her (e.g., the hungry child’s parents died when he was 1 year old) will increase the audience’s emotional reactions to the victim’s plight, thereby also increasing their willingness to donate. Indeed, workers of non-governmental charities do their best to make people want to help others. However, other studies have demonstrated that many different factors can influence *Want* itself, such as outgroup biases (Stürmer et al. 2006), prejudices against some groups (Abrams et al. 2016), and the identifiability of the person in need (Lee and Feeley 2016). It is difficult to successfully address all of these factors in any single charitable campaign. We suggest that charitable actions could be more effective when their work focuses on enhancing *Expectancy*, especially if it is difficult to evoke a desire to help. As shown here, even when desire is low, helping behavior might still be possible when expectancy is high. Therefore, our recommendation for NGO’s, or any other fundraising organization, will be to always bear in mind that, by increasing the level of expectancy of success, the potential for helping behavior is thereby increased among donating recipients. This could be achieved, for example, by assuring the recipients that the money donated will be adequately invested, and that they will eventually obtain detailed information about how their donation helped. Indeed, recent studies show that informing people about effectiveness of the help increases donations, that is, charities presented as more effective are supported more compared to those presented as less effective (Caviola et al. 2020).

Additionally, we argue that *Expectancy* is also crucial for public policy makers. Schelling (1968) pointed out that one of the biggest obstacles in effective policy decision-making is the identified victim effect, i.e., peoples’ tendency to preferentially give to identified versus anonymous victims of misfortune. He argued that, from a normative point of view, this preference for identified vs. statistical (anonymous) victim exerts a considerable impact on how public funds are spent, often leading to ineffective uses of resources in the long run. This inefficiency is based on the fact that decision-makers tend to maximize those goals which they consider to be the most valuable in the moment, as determined by affective reactions while overlooking more abstract goals, which are often of greater utilitarian value for society. We argue that increasing expectancy at this macro level (e.g., via training and workshops for health officials), can result in more utilitarian decision-making by reducing biases against prevention policies.

## Electronic supplementary material

Below is the link to the electronic supplementary material.

Supplementary file1 (DOCX 118 kb)

## Footnotes

1 In Studies 1 and 2, we manipulate the *Want* by identifiability of the victim (i.e., whether identifying information, such as the name, was present or not) in order to evoke emotions related to the victim. Since there were no effects stemming from this manipulation on any of the variables described in the paper (see Supplementary Materials), this manipulation has been omitted from further analyses. It is worth noting that the fact that manipulation was not effective does not mean that the studies are not reliable, since other researchers have also failed to confirm the identified victim effect (see meta-analyses by Hart et al. 2018; Lee and Feeley 2016). No additional manipulations or measures were included.

2 It is worth noting that *Expectancy* refers not only to the assessment of an individual’s impact on the victim, but also to the assessment of the extent to which aid will be impactful for the victim.
