# Supplementary material for: Helping when the desire is low: Expectancy as a booster
Source: Motiv Emot. 2020 Sep 4;44(6):819–31. doi: 10.1007/s11031-020-09853-3 (PMC7472690; doi:10.1007/s11031-020-09853-3)
Supplement: Supplementary file 1 — Supplementary file1 (DOCX 118 kb) [file 11031_2020_9853_MOESM1_ESM.docx]

# Additional materials

Supplementary Materials

# Supplementary Materials

**Description of victims**

**Study 1**

Identified single victim

*A 5-year old boy/ girl, Tony/ Katie, who suffers from a serious kidney condition, is now in hospital. The condition will soon lead to a kidney failure which will put the boy’s/girl’s life in danger. For medical reasons, a kidney transplant is impossible. So is hemodialysis. Recently, however, a medication which can stop the disease was discovered. Unfortunately, it is not refunded in Poland and the treatment is very expensive. If the amount of 500,000 PLN is not collected in the nearest future, the disease will be so advanced that it will be impossible to save the boy/girl.*

Non-identified single victim

*A 5-year old boy/ girl who suffers from a serious kidney condition, is now in hospital. The condition will soon lead to a kidney failure which will put the boy’s/girl’s life in danger. For medical reasons, a kidney transplant is impossible. So is hemodialysis. Recently, however, a medication which can stop the disease was discovered. Unfortunately, it is not refunded in Poland and the treatment is very expensive. If the amount of 500,000 PLN is not collected in the nearest future, the disease will be so advanced that it will be impossible to save the boy/girl.*

Identified group victim

*A group of five children, beneficiaries of a Krakow charity organization, who suffer from a serious kidney condition are now in hospital. The condition will soon lead to a kidney failure which will put the children’s life in danger. For medical reasons, a kidney transplant is impossible. So is hemodialysis. Recently, however, a medication which can stop the disease was discovered. Unfortunately, it is not refunded in Poland and the treatment is very expensive. If the amount of 500,000 PLN is not collected in the nearest future, the disease will be so advanced that it will be impossible to save the children.*

Non-identified group victim

*A group of five children who suffer from a serious kidney condition are now in hospital. The condition will soon lead to a kidney failure which will put the children’s life in danger. For medical reasons, a kidney transplant is impossible. So is hemodialysis. Recently, however, a medication which can stop the disease was discovered. Unfortunately, it is not refunded in Poland and the treatment is very expensive. If the amount of 500,000 PLN is not collected in the nearest future, the disease will be so advanced that it will be impossible to save the children.*

**Study 2**

*Dear Participant,*

*The Institute of Psychology together with the Jagiellonian University Student Volunteer Club are currently raising money to save a child/children whose story can read below.*

Non-identified group victim

*A group of five children who suffer from a serious kidney condition are now in hospital. The condition will soon lead to a kidney failure which will put the children’s life in danger. For medical reasons, a kidney transplant is impossible. So is hemodialysis. Recently, however, a medication which can stop the disease was discovered. Unfortunately, it is not refunded in Poland and the treatment is very expensive. If the amount of 500,000 PLN is not collected in the nearest future, the disease will be so advanced that it will be impossible to save the children.*

Non-identified single victim

*A 5-year old girl who suffers from a serious kidney condition, is now in hospital. The condition will soon lead to a kidney failure which will put the girl’s life in danger. For medical reasons, a kidney transplant is impossible. So is hemodialysis. Recently, however, a medication which can stop the disease was discovered. Unfortunately, it is not refunded in Poland and the treatment is very expensive. If the amount of 500,000 PLN is not collected in the nearest future, the disease will be so advanced that it will be impossible to save the girl.*

Identified single victim

*A 5-year old boy/ girl, Katie, who suffers from a serious kidney condition, is now in hospital. The condition will soon lead to a kidney failure which will put Katie’s life in danger. For medical reasons, a kidney transplant is impossible. So is hemodialysis. Recently, however, a medication which can stop the disease was discovered. Unfortunately, it is not refunded in Poland and the treatment is very expensive. If the amount of 500,000 PLN is not collected in the nearest future, the disease will be so advanced that it will be impossible to save Katie.*

*Katie used to be a happy and joyful five-year-old, living with her parents and grandmother in a small town near Krakow. Like all children, she loved spending time playing with her peers. Unfortunately, the disease that attacked suddenly made Kasia spend most of her time in the hospital under the care of doctors.*

*(A photo of a five-year old girl was used. We had the rights to use it in the study, however, we do not have the right to publish it).*

**Study 3**

Identified single victim; Low *Expectancy*

*A 5-year old boy/ girl, Tony/ Katie, who suffers from a serious kidney condition, is now in hospital. The condition will soon lead to a kidney failure which will put the boy’s/girl’s life in danger. For medical reasons, a kidney transplant is impossible. So is hemodialysis. Recently, however, a medication which can stop the disease was discovered. Unfortunately, it is not refunded in Poland and the treatment is very expensive. If the amount of 500,000 PLN is not collected in the nearest future, the disease will be so advanced that it will be impossible to save the boy/girl.*

*The money collection is ending soon and until now only 50,000 PLN was collected.*

Identified single victim; High *Expectancy*

*A 5-year old boy/ girl, Tony/ Katie, who suffers from a serious kidney condition, is now in hospital. The condition will soon lead to a kidney failure which will put the boy’s/girl’s life in danger. For medical reasons, a kidney transplant is impossible. So is hemodialysis. Recently, however, a medication which can stop the disease was discovered. Unfortunately, it is not refunded in Poland and the treatment is very expensive. If the amount of 500,000 PLN is not collected in the nearest future, the disease will be so advanced that it will be impossible to save the boy/girl.*

*The money collection is ending soon and until now already 450,000 PLN was collected.*

Non-identified group victim; Low *Expectancy*

*A group of five children who suffer from a serious kidney condition are now in hospital. The condition will soon lead to a kidney failure which will put the children’s life in danger. For medical reasons, a kidney transplant is impossible. So is hemodialysis. Recently, however, a medication which can stop the disease was discovered. Unfortunately, it is not refunded in Poland and the treatment is very expensive. If the amount of 500,000 PLN is not collected in the nearest future, the disease will be so advanced that it will be impossible to save the children.*

*The money collection is ending soon and until now only 50,000 PLN was collected.*

Non-identified group victim; High *Expectancy*

*A group of five children who suffer from a serious kidney condition are now in hospital. The condition will soon lead to a kidney failure which will put the children’s life in danger. For medical reasons, a kidney transplant is impossible. So is hemodialysis. Recently, however, a medication which can stop the disease was discovered. Unfortunately, it is not refunded in Poland and the treatment is very expensive. If the amount of 500,000 PLN is not collected in the nearest future, the disease will be so advanced that it will be impossible to save the children.*

*The money collection is ending soon and until now already 450,000 PLN was collected.*

### **Results of the manipulation of victim’s description**

**Study 1**

To test whether the manipulation of identification affected *Want* and the magnitude of donation, we ran a one-way ANOVA in which we included the condition as a factor. It turned out that there were significant differences between conditions neither on *Want*, *F* (3, 191) = 1.15, *p* = 0.33, nor the donation, *F* (3, 191) = 0.90, *p* = 0.44. The manipulation did not affect the negative affect either, *F* (3, 191) = 1.11, *p* = 0.35. *Expectancy* was also unaffected (*F* < 1).

**Study 2**

To test the effects of the identification manipulation, we run one-way ANOVAs on the variables of interest. The results showed no differences between conditions on the amount donated (logged), *F* (2, 131) = 1.85, *p* = 0.16, partial η^2^ = 0.03. However, there were differences in the number of people who decided to donate in each condition, *F* (2, 131) = 4.61, *p* = 0.012, partial η^2^ = 0.07. Bonferroni corrected pairwise comparisons showed that people donated more frequently in the identified single condition (*M* = 73%) compared to non-identified single condition (*M* = 46%). In the non-identified group condition, 69% of participants donated and that was not significantly different from the identified victim and from non-identified single victim condition (*p* = 0.053).

There was no significant effect of condition on *Want*, *F* (2, 131) = 0.72, *p* = 0.491.

**Study 3**

The manipulation of identification did not affect *Want* (F< 1). Also, there were no significant differences on negative affect; although the means followed the predicted pattern–participants in the identified condition experienced more negative emotions, *M* = 30.69 than participants in the non-identified condition did, *M* = 24.98. The difference, however, did not reach a significance level, *F* (1, 188) = 2.85, *p* = 0.093, partial η^2^ = 0.02.

|  |
| --- |

**Results for negative affect**

**Measurement of affect in Study 1 and 3**

After reading the story, participants in each condition rated their affective response. Specifically, they were asked to assess to what extent they felt each of the following emotions: 1) *nervous*, 2) *moved*, 3) *jittery*, 4) *sad*, 5) *heavy-hearted*, and 6) *depressed*, on a 0-100 scale, wherein 0 means that one does not experience a given emotion at all and 100 that a given emotion is experienced to the maximum extent. Responses to all items were averaged and used as a measure of negative affect experienced after exposure to a suffering person or group. Higher scores indicate greater negative affect.

**Study 1**

We ran a mediation analysis in which we tested the effect of negative affect on donations via *Want* and *Expectancy* (Process macro for SPSS version 2.130) (Hayes, 2013). We used 10,000 bootstrap samples and reported 95% bias corrected bootstrap confidence intervals. We expected that negative affect would be associated with *Want* (but not *Expectancy*) and would thus indirectly predict the willingness to donate. In fact, this is what we have found. Specifically, affect was associated with *Want*, *β* = 0.14, *t* = 1.92, *p* = 0.056, but not *Expectancy*, *β* = 0.02, *t* = 0.31, *p* = 0.760. Further, both *Want* and *Expectancy* were associated with the magnitude of donation, *β* = 0.55, *t* = 8.45, *p* < 0.001, for *Want* and *β* = 0.16, *t* = 2.46, *p* = 0.015, for *Expectancy*. However, affect was significantly associated with donation only via *Want*, *IE* = 0.08, SE = 0.04, 95% CI = [0.001, 0.16]. The effect of affect via *Expectancy* was non-significant, *IE* = 0.004, SE = 0.01, 95% CI = [-0.02, 0.04].

**Study 3**

In this study, we also wanted to check whether negative affect predicted donations via *Want*. Similar to Study 1, we ran mediation analyses in which we included negative affect as predictor, *Want* and *Expectancy* as parallel mediators and intention to donate (logged) as the outcome variable.

We replicated previous results showing that negative affect negatively predicted *Want*, *β* = 0.21, *t* = 2.99, *p* = 0.003, 95% CI [0.07, 0.35], but not *Expectancy*, *β* = 0.09, *t* = 1.22, *p* = 0.226, 95% CI [-0.06, 0.23], and only via *Want* it affected intentions to donate. That is, there was an indirect effect of negative affect on donation via *Want*, *IE* = 0.11, SE = 0.04, 95% CI = [0.04, 0.20]. The effect via *Expectancy* was not significant, *IE* = 0.01, SE = 0.01, 95% CI = [-0.01, 0.06].

| Table 1 | | | | |
| --- | --- | --- | --- | --- |
| *Parameters for regression equations across the studies when controlling for attitude towards money and gender.* | | | | |
| Study no. | b | *t/ Z* | *p* | CI |
| **Study 1** | | | | |
| Intercept | 0.07 | 0.92 | .470 | [-0.12,0.25] |
| Want | 0.48 | 7.25 | .000 | [0.35, 0.61] |
| Expectancy | 0.23 | 3.45 | .001 | [0.10, 0.36] |
| Expectancy × Want | -0.11 | -2.21 | .028 | [-0.20, -0.01] |
| Attitude towards money | -0.11 | -1.95 | .053 | [-0.23, 0.00] |
| Gender | -0.04 | -0.34 | .733 | [-0.27, 0.19] |
| **Study 2^a^** | | | | |
| Intercept | 1.07 | 1.96 | .050 | [0.00, 2.14] |
| Want | 0.93 | 3.35 | .001 | [0.39, 1.47] |
| Expectancy | 0.10 | 0.43 | .665 | [-0.37, 0.57] |
| Expectancy × Want | -0.53 | -1.88 | .060 | [-1.09, 0.02] |
| Attitude towards money | -0.73 | -2.90 | .004 | [-1.23, -0.24] |
| Gender | -0.25 | -0.43 | .670 | [-1.40, 0.90] |
| **Study 3** | | | | |
| Intercept | 0.43 | 2.15 | .033 | [0.04, 0.82] |
| Want | 0.48 | 6.15 | .000 | [0.32, 0.63] |
| Expectancy | 0.08 | 0.97 | .332 | [-0.08, 0.23] |
| Expectancy × Want | -0.11 | -1.88 | .062 | [-0.22, 0.01] |
| Attitude towards money | -0.10 | -1.71 | .090 | [-0.21, 0.02] |
| Gender | -0.30 | -1.93 | .055 | [-0.61, 0.01] |

^a^Parameters for logistic regression.

**Additional study (Study 0) not included in the paper**

The aim of the study is to replicate the abovementioned results using methodology applied in the previous study by Erlandsson and colleagues (2014). Additionally, we also manipulated *Want* within subjects similarly as the authors of the original study did: we provided participants with different versions of the same scenario which evoked either weaker or stronger emotions. We then checked the effects of *Expectancy* at these different levels of *Want*.

## Participants

## We have calculated a priori power analysis (G*Power 3.1) (Faul et al., 2009) with a small effect size of *f* = 0.15, the correlation among repeated measures 0.4 and power at a high level: 0.95 because of replication character of the study. It showed that a sample of at least 135 participants would be necessary to run within factor repeated measures ANOVA. We recruited 165 in case of having some low quality data. The sample comprised of volunteers approached on the university campus and who agreed to take part in the experiment. There were 102 women and 61 men (two participants did not indicate their gender) with the mean of age equal to *M* = 34.45 (*SD* = 13.43). All subjects gave informed consent before participation in the study and were debriefed on completion of the study.

Five participants who gave incomplete answers to the variables of interest were excluded from the analyses. Therefore, the final sample comprised *N* = 160 participants (with the mean of age *M* = 34.49, *SD* = 13.57; 99 were women).

## Materials and procedure

In this study, we adopted the procedure used by Erlandsson et al. (2014). Each participant was presented with four versions of a single helping situation (a full description of the procedure is presented in the Supplementary Materials). In the baseline version 1, participants were told that if they helped, the donated money would go to a Children’s village in Mozambique. In version 2, participants were told that they would sponsor a determined but unidentified child from the same village. In version 3, they were told they would sponsor an identified child (name and age were given). In version 4, they were told they would sponsor an identified child (name, age, picture, and vivid information about the child). So, version 1 was the version with the lowest *Want* to help, and version 4 was the version with the highest *Want* to help (see Table 3). Importantly, in each of the conditions, it was made clear to the participants that the money donated would go to the village as a whole and not exclusively to the sponsored child, so the objective impact of the help was always the same (see Supplementary Materials).

All four versions were summarized on one page, and participants were asked to read them. On the following pages, participants were presented with each story separately and were asked to rate their *Expectancy* and *Want* about each of them. *Expectancy* was measured with the following questions: 1) *I think one can do a lot of good*, 2) *I think it seems possible to make a big difference*, 3) *I believe the expected consequences are very positive*. *Want* was measured with two added items: 1) *It is important to me to help in this satiation* and 2) *I want to help in this situation*. Although it was not our main interest, we also included the other measures used in the original study: personal distress, sympathy, and perceived responsibility. The results for these additional measures are presented in the Supplementary Materials. Answers to all items were given on 1-7 scale anchored with *definitely disagree* to *definitely agree.*

On the last page, participants were told they had 100 PLN they could use to help and asked to distribute the amount between the four versions. They were instructed that they could divide this amount freely in the closest way possible to what they would do in real life. The amount donated in each version was used as the dependent variable.

## Results and Discussion

Descriptive statistics are presented in Table 0.

### Preliminary analyses

To analyze our data, we used a similar approach to that employed by Erlandsson et al. (2014). Thus, we compared version 4 with version 1. In the original analysis, the authors used difference scores to test within-subjects mediation effects. We, however, applied the MEMORE macro for SPSS which helped us perform similar analyses (Montoya & Hayes, 2017). MEMORE (MEdiation and MOderation analysis for REpeated measures designs) is an approach that allows testing several parallel mediation effects on the data coming from repeated measures.

In our model, change in *Want* and *Expectancy* between version 4 and 1 were the mediators and change in the magnitude of donation served as the outcome variable. Changes in all variables were statistically significant: b = 13.59, *t* = 3.06, *p* = 0.003, *95% CI* [4.83, 22.34], for donation; b = 0.65, *t* = 7.12, *p* < 0.001, *95% CI* [0.47, 0.83], for *Want*; and b = 0.47, *t* = 5.84, *p* < 0.001, *95% CI* [0.31, 0.63], for *Expectancy*.

### Want × Expectancy Interaction

Since in this study we utilized a repeated measures design, we could not repeat the same interactional analyses we ran in the previous studies. Therefore, to test the effects of *Expectancy* on donation at different levels of *Want*, we checked the relationships between *Expectancy* and donations in conditions in which *Want* was lowest and highest.

As shown in Table 0, *Want* was the lowest in version 1 and version 2, with the two conditions not significantly differing from each other. The highest *Want* was in version 4. We thus checked the correlations between *Expectancy* and donation is these conditions. It was equal to *r* = .18, *p* = .020 for version 1, *r* = .27, *p* = .001 for version 2, *r* = .19, *p* = .015 for version 3, and *r* = .07, *p* = .375 for version 4. The results thus show that when the *Want* is highest (in the last condition), *Expectancy* is not a significant predictor. However, when the *Want* is lower, *Expectancy* plays a significant role.

Further, we checked whether the differences between these relationships were significant. To that aim, we compared correlations between *Expectancy* and donation from the two conditions with the highest and lowest *Want*: *r* = .27 for version 2 and *r* = .07 version 4. We used the cocor R package (Diedenhofen & Musch, 2015) with a test of two overlapping correlations based on dependent groups. The comparisons showed that the difference between the two coefficients was significant, Pearson and Filon’s *z* = 1.72, *p* = 0.042.

The results thus show that *Expectancy* played a different role depending on the level of *Want*. It played a significant role only when the *Want* was low and did not matter when the *Want* was high, just as in the previous studies. The results of this study are important in that they replicate the findings of the earlier studies while doing so within the framework of completely different experimental materials and a different experimental design.

| Table 0 | | | | | | | | |
| --- | --- | --- | --- | --- | --- | --- | --- | --- |
| *Descriptive statistics for variables tested in Study 0 (N = 160).* | | | | | | | | |
|  | statistical victim | | determined victim | | identified victim | | vivid identified victim | |
|  | *M* | *SD* | *M* | *SD* | *M* | *SD* | *M* | *SD* |
| 1. Want [1-7] | 4.59 | 1.39 | 4.54 | 1.34 | 4.84 | 1.35 | 5.27 | 1.37 |
| 2. Expectancy [1-7] | 4.91 | 1.24 | 4.90 | 1.12 | 5.08 | 1.12 | 5.39 | 1.18 |
| 3. Money donated [1-100 PLN] | 25.90 | 30.91 | 12.38 | 12.44 | 19.56 | 20.91 | 39.28 | 31.48 |

**Materials presented to participants in Study 0**

For over 60 years, SOS Children’s Villages has helped children in need. Today we run children’s villages in 133 countries where orphan or abandoned children can get a new home. In everything we do, cost effectiveness is highly important as we want your money to go to the ones who need it the most – the children. In 2011, 85.4 % of our received money went to the villages. Our administration and marketing costs were only 14.6 %.

**Version 1:** If you choose to support us today, you will be the sponsor of a children’s village in Mozambique. Your money will go exclusively to food, clothes and education for all children living in the village that you sponsor.

**Version 2:** If you choose to support us today, you will be the sponsor of a specific child in one of our children’s villages in Mozambique. The child you sponsor is anonymous so you will not know its identity. Your money will go exclusively to food, clothes and education for all children living in the village where your sponsored child lives.

**Version 3:** If you choose to support us today, you will sponsor Isaka who lives in one of our children’s villages in Mozambique. Isaka is a five year old boy and lives in Tete in western Mozambique. Your money will go exclusively to food, clothes and education for all children living in the village where Isaka lives.

**Version 4**: If you choose to support us today, you will sponsor Isaka who lives in one of our children’s villages in Mozambique. Isaka is a five year old boy and lives in Tete in western Mozambique. Your money will go exclusively to food, clothes and education for all children living in the village where Isaka lives.


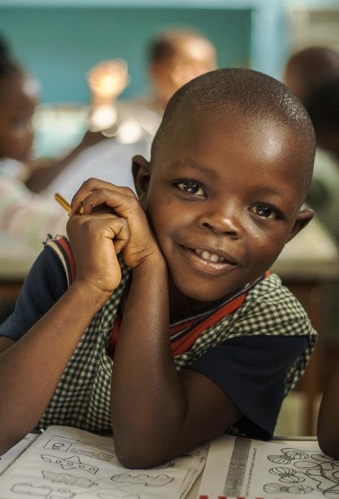


Isaka Charles Dominick

Born on 16th April, 2012

His mother died when he was 1 year old and that he lived with his grandmother until she could not take care of him anymore. After coming to the children’s village, he is no longer malnourished and does well in pre-school, but still has occasional nightmares.

# 
